# Association of Cord Blood Glucose, Sodium, Potassium, and Calcium Levels With Neonatal Birth Asphyxia: A Hospital-Based Study

**DOI:** 10.7759/cureus.26115

**Published:** 2022-06-20

**Authors:** Anand K Pyati, Pradeep K Khanikekar, Nagaraj R Shetkar, Mallanagouda M Patil, Purushottam B Jaju, Madhu Latha Karra, Sudharani A Pyati, Mohd Shannawaz

**Affiliations:** 1 Biochemistry, All India Institute of Medical Sciences, Bibinagar, Hyderabad, IND; 2 Family Medicine, Indian Army Military Hospital, Secunderabad, IND; 3 Biochemistry, Khaja Bandanawaz University (KBN) Institute of Medical Sciences, Kalaburagi, IND; 4 Pediatrics, BLDE (Deemed to be University) Shri. B. M. Patil Medical College, Hospital and Research Centre, Vijayapura, IND; 5 Obstetrics and Gynecology, BLDE (Deemed to be University) Shri. B. M. Patil Medical College, Hospital and Research Centre, Vijayapura, IND; 6 Pedodontics and Preventive Dentistry, Pyati Pedodontics Hospital, Hyderabad, IND; 7 Biostatistics and Epidemiology, Amity Institute of Public Health, Noida, IND

**Keywords:** calcium, potassium, sodium, glucose, apgar score, asphyxia neonatorum

## Abstract

Context

Neonatal birth/perinatal asphyxia is a serious condition with the potential to cause damage to various tissues of the body especially the brain. Hypoxia can cause metabolic disturbances, which in turn can lead to imbalances in the levels of glucose, electrolytes, and calcium, which can further worsen the condition. Early detection of these biochemical derangements and immediate correction can prevent the complications and lifelong disabilities of birth asphyxia due to injury to vital organs particularly the brain. The aim is to assess any correlation between the cord blood glucose, electrolytes, and calcium levels and the severity of birth asphyxia.

Methods and material

In this study, 50 birth asphyxia neonates with birth weight >2.5 kg, and a 5-minute Apgar score ≤ 6 at birth with clinical evidence of asphyxia were compared with healthy neonates with birth weight > 2.5 kg, and a 5-minute Apgar score > 7. In all the cases and controls, cord blood glucose was estimated by glucose oxidase and peroxidase (GOD-POD) method, total calcium by Arsenazo method, and sodium and potassium were estimated by ion-selective Electrode (ISE) method using fully automated biochemistry analyzers.

Results

The mean cord blood concentrations of glucose, sodium, potassium, and calcium were significantly lower among birth asphyxia neonates in comparison with that of controls (p < 0.05). The correlation coefficient (r) for the study variables among cases indicates that there is a low to moderate positive correlation between the 5-minute Apgar score which is a measure of severity of birth asphyxia and cord blood concentrations of glucose, sodium, and calcium.

Conclusion

In our study, birth asphyxiated neonates were found to have statistically significant low levels of cord blood glucose and electrolytes like sodium and calcium except for potassium. There was a low to moderate positive correlation between cord blood glucose and electrolyte concentrations with the severity of birth asphyxia. Analysis of cord blood for these simple biochemical tests can help pediatricians in the active management of birth asphyxia cases.

## Introduction

Birth asphyxia is characterized by “the failure to establish breathing at birth” [1]. The National Neonatology Forum of India defines birth asphyxia as “gasping and ineffective breathing or lack of breathing at one minute after birth” [2]. Inadequate supply of oxygen which is seen in perinatal asphyxia when severe can cause hypoxic-ischemic damage to all the organs, particularly kidneys (50%), brain (28%), heart (25%), lungs (23%), liver and intestine in neonates which can lead to major permanent complications including death [3]. Cerebral palsy, renal cortical necrosis, persistent pulmonary hypertension, hypotension, cardiogenic shock, or heart failure are the commonly encountered complications among the survivors [4].

Birth asphyxia is a common neonatal health problem and contributes significantly to neonatal morbidity and mortality. Worldwide, every year 4 million deaths among newborns and 3.2 million stillbirths occur and out of which, perinatal hypoxia contributes 23% and 29%, respectively. Around 1 million children who survive after being affected by birth asphyxia, continue to suffer from long-lasting neurological and developmental disabilities [5]. In the Indian scenario, 28.8% of the deaths among newborns and 45.5% of the stillbirths are attributed to perinatal asphyxia [6]. Around eight and two newborns for every hundred newborns in India will have an Apgar score of < 7 at one and 5 minutes after birth, respectively [6,7]. Due to lack of resources, developing countries are worst affected. Yet, it is an eminent global health issue warranting urgent attention.

APGAR score is the most convenient and commonly used tool for the evaluation of asphyxia in neonates. Five elements, the APGAR score considers are color, heart rate, reflexes, muscle tone, and respiration, each of which is given a score of 0, 1, or 2. It is reported at 1 minute and 5 minutes after birth and the total score can range from 0 to 10. In a term neonate, a 5-minute Apgar score of 7-10 is interpreted as reassuring, a score of 4-6 as moderately abnormal, and a score of 0-3 as low [8]. Several studies have shown a significant correlation between low Apgar scores and poor health outcomes in the survivors [9].

In a biological system, sodium, potassium, and calcium are the most important electrolytes, and any significant fluctuations in the blood concentrations can lead to metabolic derangements causing convulsions and shock. Calcium has many crucial functions in the body such as acting as an important second messenger, required for normal muscle contraction and many enzyme reactions. Tight regulation and maintenance of normal blood concentrations of these electrolytes are important for optimal functioning of the body [10].

Assessment and management of electrolyte status in the newborn is a very crucial and difficult task. Water and electrolyte levels in the body can vary largely during the transition from fetal to newborn life. Before birth, the fetus receives the nutrients including fluid and electrolytes from the maternal blood and their levels are predominantly controlled by the maternal regulatory system. After the birth, the neonate should quickly take over the charge of water and electrolyte balance independently and efficiently in an outside unfavorable environment. Therefore, transitory alterations in the fluid and electrolyte levels can be expected and even a minor change in the absolute concentrations of these electrolytes can suggest proportionately substantial variation for the newborn considering its relatively small size [11].

In 90% of term babies, the asphyxia occurs during antepartum and/or intrapartum period. The important maternal/fetal conditions associated with perinatal asphyxia are eclampsia, diabetes mellitus, Rh isoimmunization, antepartum hemorrhage, renal or pulmonary disease, cephalopelvic disproportion, IUGR, meconium-stained liquor, prolonged labor [12].

An adequate amount of oxygen is required for the cellular metabolic processes. During perinatal hypoxia, there is a shift in the metabolic process from aerobic to anaerobic glycolysis, wherein the energy yield will be less and increased production of lactic acid which can lead to metabolic acidosis. When the hypoxia is prolonged, the cardiac output falls and blood flow to the brain, kidney, and other vital organs may be compromised leading to hypoxic-ischemic organ damage. The blood glucose levels may fall due to increased utilization of glucose for anaerobic glycolysis. Serum potassium levels may be high because of increased cellular damage, which can further lead to lower serum sodium levels due to increased secretion of ADH and water retention. Serum calcium levels tend to drop due to hypoxic-ischemic damage to parathyroid glands [4].

Earlier studies have found a higher incidence of hypoglycemia, low sodium and calcium levels, and high potassium levels in asphyxiated neonates as compared to healthy controls [4,13-17]. Most of those studies have been done in blood samples of neonates collected at 24-48 hours after birth. But there are not many studies on cord blood for assessing the risk of hypoxic-ischemic tissue damage in the early neonatal period.

Despite increasing knowledge regarding the underlying mechanisms for birth/perinatal asphyxia, early detection of hypoxic-ischemic organ damage remains challenging in neonatal care. Hence, the purpose of this study was to determine any association between the cord blood glucose, electrolytes, and calcium levels and the severity of birth asphyxia, so that complications related to hypoxic-ischemic injury can be anticipated early and appropriate treatment can be initiated at the earliest to reduce the neonatal morbidity and mortality.

Objectives

The first is to estimate the cord blood concentrations of glucose and electrolytes namely sodium, potassium, and calcium in asphyxiated neonates and compare them with those of normal non-asphyxiated control newborn babies. The second is to ascertain any correlation between cord blood concentrations of glucose, sodium, potassium, and calcium with a 5-minute Apgar score which is a measure of severity of birth asphyxia in both asphyxiated and non-asphyxiated neonates.

## Materials and methods

Study design

This is a hospital-based cross-sectional study.

Study setting

Department of Obstetrics and Gynecology, Department of Pediatrics and Department of Biochemistry, BLDE (Deemed to be University) Shri. B. M. Patil Medical College, Hospital and Research Centre, Vijayapura, Karnataka during the period from January 2012 to December 2014.

Sample size

N=100; n=50 full term (>37 weeks) birth asphyxia neonates, n=50 full term (>37 weeks) healthy newborn babies.

Prior to the commencement of the study clearance from the Institutional Ethical Committee was obtained (Institutional Ethical Committee, BLDE [Deemed to be University] Shri. B. M. Patil Medical College, Hospital and Research Centre, Vijayapur, approval no. 39/2012). Guidelines of the revised (2013) Helsinki Declaration of 1975 were followed. Before recruitment of cases and controls, the pregnant women/parents/guardian of all the cases and controls have explained the purpose of the study in their local language, and their written informed consent was obtained. Trained obstetricians conducted deliveries and all the neonates were duly taken care of by the neonatologists.

The full-term (37 to 40 weeks of gestation) pregnant mothers aged between 20 and 45 years who are in the first stage of labor under the supervision of a trained & experienced obstetrician and pediatrician were closely monitored during and after delivery until discharge. The data regarding socio-demographic characteristics including age, parity, and literacy, maternal risk factors such as the history of diabetes, hypertension, stillbirths, infections, anemia, etc., presence of antenatal risk factors such as antepartum hemorrhage, pregnancy-induced hypertension, premature rupture of membranes and information regarding intrapartum events such as induction of labor, prolonged labor, meconium staining of amniotic fluid, presentation, cord around the neck instrumental delivery and cesarean section and neonatal particulars like sex, birth weight of the baby were noted in a predesigned proforma. An experienced pediatrician and neonatologist assessed the neonates at 1 minute and 5 minutes after birth for heart and respiratory rates, muscle tone, reflexes, and color, and Apgar score was calculated. Later, a detailed clinical and neurological examination was done to detect any clinical signs of hypoxic-ischemic encephalopathy (HIE).

Neonates with birth weight >2.5 kg, and Apgar score ≤ 6 at 5 minutes of birth with minimum two or more of the following criteria such as blood pH ≤ 7.2, abnormal fetal heart rate (<100/min or > 160/min) and/or meconium-stained amniotic fluid, seizures, and/or coma or those who required resuscitation with >1 min of positive pressure ventilation were considered as birth asphyxia cases. Controls were those neonates with birth weight > 2.5kg, and Apgar score ≥ 7 at 5 minutes of birth with normal fetal heart rate and clear amniotic fluid. Neonates having birth anomalies, suspected inborn errors of metabolism, born to mothers who were on diuretics/anti-hypertensives/hypoglycemic drugs or had a history of pregnancy-induced hypertension or eclampsia, lower section cesarean section (LSCS) on General anesthesia or on drugs such as phenobarbitone, pethidine, MgSO4 and any such medications which can lead to neonatal respiratory distress and history of maternal pyrexia within two weeks prior to delivery were excluded from the study.

From all the cases and controls, after Inclusion in the study as per the above-mentioned inclusion and exclusion criteria, 2 mL of cord blood samples were drawn using all sterile precautions in an appropriate container. A blood sample was subjected to centrifugation half an hour after collection and serum was isolated and used for study investigations immediately. Cord blood serum level of glucose was estimated by glucose oxidase and peroxidase (GOD-POD) method [18] and total calcium by Arsenazo [18] method using Cobas c 311 fully automated clinical chemistry analyzer (Roche Diagnostics International Ltd.). Cord blood serum levels of electrolytes (sodium and potassium) were estimated by ion-selective electrode (ISE) method [19], using Acculite-3P fully automated electrolyte analyzer (Rapid Diagnostic Pvt. Ltd., Delhi).

Statistical analysis

The data obtained from the present study were analyzed using Statistical Package for the Social Sciences statistical software (SPSS) for Windows (Version 21, IBM Corp., Armonk, NY, USA). Continuous variables are expressed as mean ± Standard deviation (SD) and range values and categorical data are presented as percent frequency of occurrence. Two-tailed unpaired t-test was used to compare the difference between the means of the two study groups. Association between two variables was assessed using Pearson’s correlation coefficient for parametric variables and Spearman’s coefficient for non-parametric variables. P-values of ≤0.05 were treated as statistically significant.

## Results

Table 1 shows the demographic and pregnancy-related details of asphyxiated neonates and controls. All the cases and controls were full term neonates weighing more than 2.5 kg at birth. Of the 100 neonates included in the study, 57 were male and 43 were female babies. Asphyxia was more common among male babies (58%) than in comparison to females (42%). The majority of the cases were delivered by normal vaginal delivery (60%). 70% of the asphyxiated babies are born to primigravida mothers. Among the 50 hypoxia cases, 34 (68%) developed HIE. Of the 34 HIE cases, 17 (50%) were stage I, 13 (38.2%) were stage II, and four (11.7%) were stage III encephalopathy cases.

**Table 1:** Demographics, pregnancy and delivery details, and sequelae of cases and controls LSCS = Lower Segment Cesarian Section, HIE = Hypoxic Ischemic Encephalopathy

Variable	Cases n (%)	Controls n (%)	P-value
Gender			
Male	29 (58)	28 (46)	0.840
Female	21 (42)	22 (44)	
Birth weight (kg)			
2.5 – 3.5	47 (94)	49 (98)	0.307
> 3.5	3 (6)	1 (2)	
Mode of delivery			
Normal	30 (60)	38 (76)	0.208
LSCS	18 (36)	12 (24)	
Instrumental	2 (4)	0 (0)	
Parity			
Primi	35 (70)	38 (76)	0.499
Multi	15 (30)	12 (32)	
Gestation age (Weeks)			
37 - 40	50 (100)	50 (100)	-
Sequalae			
HIE	34 (68)	0 (0)	-

Table 2 displays the comparison of Apgar score and mean cord blood concentrations of glucose, and electrolytes among the neonates with and without birth asphyxia. The mean Apgar score and cord blood concentrations of glucose, sodium, potassium, and calcium were lower among birth asphyxia neonates in comparison with that of controls. Unpaired student’s t-test analysis showed statistically considerable change (p<0.05) in the levels of above-mentioned study variables between the two study groups except for potassium.

**Table 2:** Comparison of Apgar score and serum levels of glucose, sodium, potassium and calcium in neonatal birth asphyxia cases and controls APGAR = Appearance, Pulse, Grimace, Activity, and Respiration P-value of ≤ 0.05 = Statistically significant

	Cases (n=50) (Mean ± SD)	Controls (n=50) (Mean ± SD)	P-value
Apgar score	4.4 ± 1.03	7.9 ± 0.99	< 0.001
Glucose (mg/dL)	35.03 ± 4.14	83.4 ± 23.8	<0.001
Sodium (mEq/L)	133.3 ± 8.6	138.3 ± 6.7	0.002
Potassium (mEq/L)	4.7 ± 0.6	4.6 ± 0.7	0.445
Calcium (mg/dL)	8.5 ± 1.6	9.6 ± 0.2	< 0.001

Table 3 depicts linear statistical association between Apgar score and cord blood levels of glucose and electrolytes in study among the neonates with and without birth asphyxia. The correlation coefficient (r) for the study variables among cases indicates that, there is a low to moderate positive correlation between the 5-minute Apgar score which is a measure of severity of birth asphyxia and cord blood concentrations of glucose, sodium, and calcium. But as indicated by p-values, correlation coefficient is statistically not significant, which may be because of the non-linear relationship between the study variables.

**Table 3:** Statistical association between Apgar score and cord blood levels of glucose and electrolytes in study among the neonates with and without birth asphyxia APGAR = Appearance, Pulse, Grimace, Activity, and Respiration P-value of ≤ 0.05 = Statistically significant

Variables	APGAR SCORE			
	Cases		Controls	
	r value	P-value	r value	P-value
Glucose	0.21	0.586	0.14	0.654
Sodium	0.05	0.451	0.32	0.256
Potassium	0.10	0.523	-0.18	0.365
Calcium	0.005	0.751	0.025	0.874

## Discussion

Neonatal birth/perinatal asphyxia is a serious condition with the potential to cause damage to various tissues of the body especially the brain which is more prone to hypoxic-ischemic injury. Hypoxia can cause metabolic disturbances, which in turn can lead to imbalances in the levels of glucose, electrolytes, and calcium, which can further worsen the condition. Early detection of these biochemical derangements and immediate correction can prevent the complications and lifelong disabilities due to brain injury, renal cortical necrosis, etc. So early diagnosis and timely fluid and electrolyte management are required for a better outcome in neonates.

In the present study, the cord blood glucose, sodium, potassium, and calcium levels were assessed among 50 asphyxiated and 50 normal-term neonates whose birth weight was 2.5 kg or more. Birth asphyxia was more common among male neonates and also those born by normal/instrumental delivery when compared to females and the caesarian section, though the difference is not statistically significant. Among the 50 hypoxia cases, 34 (68%) developed HIE (Table 1). Of the 34 HIE cases, 17 (50%) were stage I, 13 (38.2%) were stage II, and four (11.7%) were stage III encephalopathy cases. Among the 34 HIE cases, 23 (67.6%) were normal, nine (26.5%) developed neurological abnormalities, and two babies (5.9%) died.

In our study, we used a cord blood sample immediately after birth for biochemical analysis in contrast to the venous blood sample at 24-48 hours after birth as in most of the previous studies. Analysis of the cord blood has the advantage of detecting the electrolyte and other biochemical derangements at the early stage before the clinical/radiological signs appear or the irreversible damage sets in [16].

The mean cord blood glucose concentration in birth asphyxia cases was considerably lower in our study (35.03 ± 4.14 mg/dL) when compared to controls (83.4 ± 23.8 mg/dL) (p < 0.001) (Table 2). Considerably low cord blood glucose was found in 43.3% of birth asphyxia cases. A positive correlation was observed between the cord blood glucose concentrations and the 5-minute Apgar score among the cases (Table 3). These observations are consistent with Kavya et al. [4], Basu et al. [10], Sasidharan et al. [13], Bahatkar et al. [16], Islam et al. [20], Joag et al. [21], Rai et al. [22], and Kumar et al. [23].

During the first 60-90 minutes after delivery, the blood glucose concentration in neonates decreases drastically. But the counter regulatory mechanisms will again normalize the glucose levels by activation of glycogenolysis, gluconeogenic and lipolytic pathways. Neonates with birth asphyxia may have hypoglycemia due to impaired gluconeogenesis and exhaustion of glycogen stores because of associated excess of catecholamines during the perinatal period. Whereas hypoglycemia seen in premature and IUGR babies can be attributed to limited reserves of glycogen, babies born to mothers with gestational diabetes are in danger due to hyperinsulinemia despite having increased glycogen stores [11].

In our research, the mean cord blood sodium concentration in birth asphyxia cases was substantially lower (133.3 ± 8.6 mEq/L) when compared to controls (138.3 ± 6.7 mEq/L) and the difference was statistically significant (p = 0.002) (Table 2). A significantly low level of cord blood sodium was seen in 53.3% of patients. A positive correlation was found between the cord blood glucose concentrations and the 5-minute Apgar score among the cases (Table 3). Such results are in line with previous research of Kavya et al. [4], Basu et al. [10], Gupta et al. [14], Bahatkar et al. [16], Manjunatha Babu et al. [17], Islam et al. [20], Joag et al. [21], Kumar et al. [23], Acharya et al. [24], Hasan et al. [25], and Thakur et al. [26]. Hyponatremia seen in birth asphyxia cases may be attributed to the increased water retention and fluid overload either because of renal insufficiency or hypersecretion of ADH [4].

The mean cord blood potassium concentration was slightly higher in birth asphyxia babies (4.7 ± 0.6 mEq/L) in comparison with the normal babies (4.6 ± 0.7 mEq/L). However, statistically, the difference between the two groups was not significant (p = 0.445) (Table 2). Earlier, the research studies by Kavya et al. [4], Basu et al. [10], Gupta et al. [14], Bahatkar et al. [16], and Kumar et al. [23] also found similar results. Whereas Manjunatha Babu et al. [17], Islam et al. [20], Acharya et al. [24], Hasan et al. [25], and Thakur et al. [26] found statistically significant elevation of serum potassium levels among asphyxia cases when compared to controls. Hypoxia due to birth asphyxia can cause acidosis leading to the movement of intracellular K+ ions to form RBCs into the plasma in exchange for H+ ions, thus raising the concentration of potassium in blood [27]. Further, hypoxic-ischemic cellular damage to various other tissues can also raise serum potassium levels [4]. Also, acute renal failure secondary to asphyxia can lead to higher potassium levels due to decreased excretion [16].

There was a significant reduction in the mean cord blood calcium concentration among birth asphyxia babies (8.5 ± 1.6 mg/dL) in comparison with their control counterparts (9.6 ± 2.2 mg/dL) (p < 0.001) (Table 2). Lower cord blood calcium was found in 23.3% of asphyxia cases. A positive correlation was found between the cord blood calcium concentrations and 5-minute

Apgar scores among the cases (Table 3). Similar results were observed in earlier research studies of Kavya et al. [4], Basu et al. [10], Bahatkar et al. [16], Manjunatha Babu et al. [17], Islam et al. [20], Joag et al. [21], Rai et al. [22], and Kumar et al. [23]. Normally, the stimulation of parathyroid hormone secretion and consequent mobilization of calcium from bones normalizes the decreasing blood calcium levels in neonates immediately after birth. Significantly lower calcium levels in the cord blood and the neonates can be because of the sluggish response of the parathyroid glands secondary to perinatal hypoxia [4,11]. The non-significant ‘p’ values for the correlation studies for cord blood glucose and electrolyte concentrations with Apgar score in our study may be because of a non-linear relationship between the variables.

Limitations

Intravenous fluid and oxytocin used at the time of delivery were not taken into account, which might affect the electrolyte status in the mother and hence in the cord blood.

## Conclusions

It is critical to diagnose and treat newborns with birth asphyxia as early as possible in order to avoid lasting neurological and developmental disabilities. In our study, birth asphyxiated neonates were found to have statistically significant low levels of cord blood glucose and electrolytes like sodium and calcium except for potassium. There was a low to moderate positive correlation of cord blood glucose and electrolyte concentrations with the severity of birth asphyxia. Analysis of cord blood for these simple biochemical tests can help pediatricians in the active management of birth asphyxia cases. Further research with a large sample size is required to ascertain the cause-effect relationship between the study variables and the disease.
